# Giant voltage-induced modification of magnetism in micron-scale ferromagnetic metals by hydrogen charging

**DOI:** 10.1038/s41467-020-18552-z

**Published:** 2020-09-24

**Authors:** Xinglong Ye, Harish K. Singh, Hongbin Zhang, Holger Geßwein, Mohammed Reda Chellali, Ralf Witte, Alan Molinari, Konstantin Skokov, Oliver Gutfleisch, Horst Hahn, Robert Kruk

**Affiliations:** 1 Institute of Nanotechnology, Karlsruhe Institute of Technology, 76344 Eggenstein-Leopoldshafen, Germany; 2 Institute of Materials Science, Technische Universität Darmstadt, 64287 Darmstadt, Germany; 3 Institute for Applied Materials, Karlsruhe Institute of Technology, 76344 Eggenstein-Leopoldshafen, Germany; 4 Present Address: IBM Research – Zurich, 8803 Rüschlikon, Switzerland

**Keywords:** Magnetic properties and materials, Ferromagnetism

## Abstract

Owing to electric-field screening, the modification of magnetic properties in ferromagnetic metals by applying small voltages is restricted to a few atomic layers at the surface of metals. Bulk metallic systems usually do not exhibit any magneto-electric effect. Here, we report that the magnetic properties of micron-scale ferromagnetic metals can be modulated substantially through electrochemically-controlled insertion and extraction of hydrogen atoms in metal structure. By applying voltages of only ~ 1 V, we show that the coercivity of micrometer-sized SmCo_5_, as a bulk model material, can be reversibly adjusted by ~ 1 T, two orders of magnitudes larger than previously reported. Moreover, voltage-assisted magnetization reversal is demonstrated at room temperature. Our study opens up a way to control the magnetic properties in ferromagnetic metals beyond the electric-field screening length, paving its way towards practical use in magneto-electric actuation and voltage-assisted magnetic storage.

## Abstract (web summary)

“Electric field control of ferromagnetism is typically limited by screening, and is restricted to the first few layers of metals. Here the authors overcome this limitation via the absorption of hydrogen into metal structure, demonstrating voltage control of magnetic properties of micron-scale SmCo5.”

## Introduction

Controlling magnetic properties of magnetic materials by applying small voltages have attracted great interests owing to its ultralow power consumption^1–3^. Most studies so far have been conducted at low temperatures using diluted magnetic semiconductors^4^ and multiferroics^5^. By contrast, ferromagnetic metals and intermetallic compounds exhibit high Curie temperatures and large magnetization, making the voltage control of their magnetic properties desirable for room-temperature applications. However, unlike semiconductors and multiferroics, the metallic materials have strong electric-field screening, which makes the voltage control of their magnetic properties difficult. The breakthrough was made by Weisheit and colleagues, who showed that the coerciviy of ultrathin films of FePt(Pd) can be tuned by ~0.005 T by applying voltages to change the electron density at the metal/electrolyte interface^6^. The success of this experiment took advantage of the large surface-to-volume ratio of ultrathin film and the ultrahigh electric field in electrochemical double layer (>10^9^ V m^−1^). However, due to strong electric-field screening, the modification of magnetic properties by this charge-doping method is restricted to a few atomic layers^6–9^. In addition, the changes in magnetic properties are too small for practical use^6,7,9^.

Recently, magneto-ionics have been employed to control the magnetic properties of ultrathin metal films. Compared with the charge-doping method, magneto-ionics use ions such as O^2−^ and H^+^, instead of electrons/holes, as the tuning agent^10–14^. For instance, in the Co (0.9 nm)/GdO_x_ system^11,12^, O^2−^ ions in the ionic-conducting GdO_x_ layer, driven by the electric field, migrate towards/away from the GdO_x_/Co interface. The change of oxidation state and crystal structure in the ultrathin Co layer permits to modify its magnetic properties. However, due to electric-field screening in the metal layer, tuning of magnetism via ionic migration is generally limited to the interfacial region within a few atomic layers^10–13^. Although tuning of metallic layer with larger thickness (~15 nm) has also been achieved by magneto-ionics, these tuning processes often suffer from the inherent irreversibility, typical of electrochemical conversion-type reactions^15^. Moreover, the modulation of coercivity reaches only a few tens of mT, thus hindering the practical use of voltage-tuning effect^11,12^. Hence, tuning of the magnetic properties in the volume of ferromagnetic metals by small voltages strong enough from a practical point of view and fully reversible at the same time still remains a challenge.

One, yet unexplored, approach to overcome the electric-field screening limitations is through the insertion and extraction of hydrogen atoms in the metal structure. In the 1970s studies show that some metal and their intermetallic compounds can absorb large amounts of hydrogen atoms that act as hydrogen-storage materials^16,17^. In contrast to electrons and ions, hydrogen atoms are electrically neutral, and therefore their diffusion into the metal structure is not restricted by electric-field screening, offering the opportunity to overcome the limitations of the electric-field screening length in ferromagnetic metals. Moreover, the incorporation of hydrogen atoms often involves the distortion of crystal structure and change of electronic structure, which may change magnetic properties^18,19^. In these studies, however, the absorption of hydrogen atoms was carried out in hydrogen gas usually at high temperature and with high hydrogen pressures. In order to realize the tuning of magnetic properties with small voltages, it would be desirable if the absorption and desorption of hydrogen atoms could be controlled by electrochemical potentials, as established in nickel-metal hydride batteries^19^. Thermodynamically, the hydrogen pressure at certain temperatures can be converted into electrochemical potentials through Nernst equation, which in principle makes the electrochemically controlled hydrogen charging/discharging possible. To test the idea, we selected SmCo_5_ as a model material based on two criteria. Firstly, its equilibrium hydrogen pressure is 4 atm at room temperature^15^ and according to Nernst equation^20^, it is calculated that the equivalent electrochemical potential is only ~17 mV more negative than the standard water electrolysis potential, making SmCo_5_ suitable for voltage-controlled hydrogen charging/discharging. Secondly, SmCo_5_ is widely used as an important permanent magnet for its large coercivity, large magnetization (100 A m^2^ kg^−1^), and high Curie temperature (1020 K)^21^ and is considered candidate material in next-generation ultrahigh density magnetic storage (area density ~10 TB per square inches) because of its exceptionally high magnetocrystalline anisotropy (~17.2 MJ m^−3^)^22^. Tuning of its magnetic properties, particularly magnetocrystalline anisotropy and coercivity with small voltages, would create novel magneto-electric functions in the context of applications.

Here, using micrometer-sized SmCo_5_ powder, we show that it is possible to reversibly charge and discharge the material with hydrogen atoms by applying small voltages. Employing this approach, the coercivity of SmCo_5_ powder is tuned by ~1 T, more than two orders of magnitudes larger than previously achieved in ultrathin films by charge doping^6,7,9^ and magneto-ionics^10–13^. This enables voltage-assisted magnetization reversal in high-anisotropy SmCo_5_ at room temperature.

## Results

### Electrochemically controlled charging and discharging with hydrogen atoms

**Fig. 1:**
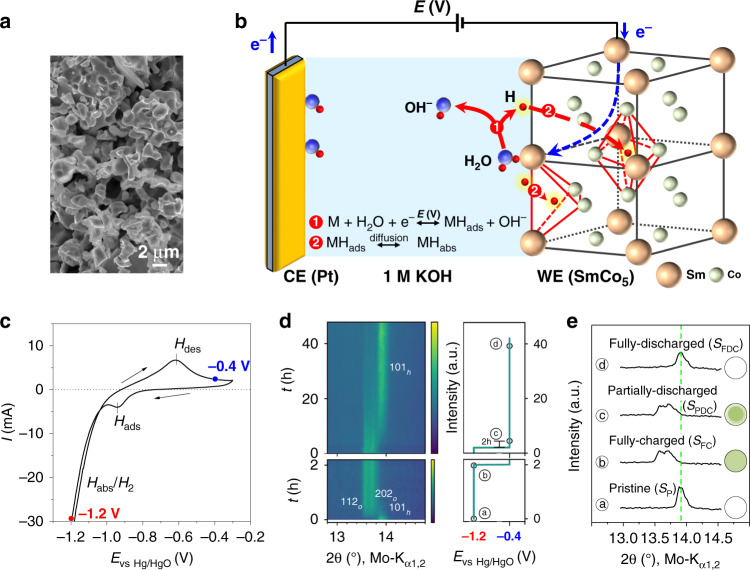
Voltage-controlled charging and discharging of SmCo_5_ with hydrogen atoms. **a** An SEM micrograph of the SmCo_5_ powder, showing particle sizes in the micrometer range. **b** Schematic of the voltage-controlled charging and discharging of hydrogen atoms in the crystal structure of SmCo_5_. WE working electrode; CE counter electrode. Hydrogen atoms originate from the electrochemical reduction of water molecules at the metal/electrolyte interface (reaction ①). They first adsorb onto the metal surface (*H*_ads_), and then, driven by the concentration gradient, diffuse into the octahedral or tetrahedral interstitial sites (*H*_abs_, reaction ②). For clarity, the extraction process is not depicted. **c** A cyclic voltammogram curve of the SmCo_5_ electrode in 1 M KOH with a scan rate of 2 mV s^−1^. The voltage is with respect to Hg/HgO electrode. **d** Contour plot of in situ XRD patterns of the SmCO_5_ electrode under the sequential voltages of −1.2 V and −0.4 V. **e** XRD patterns for the samples indicated in **d** with different charging states, i.e. as-prepared (ⓐ, *S*_P_), fully charged (ⓑ, *S*_FC_), partially discharged (ⓒ, *S*_PDC_) and fully discharged (ⓓ, *S*_FDC_). The inset schematically illustrates the cross sections of the SmCo_5_ particle at these charging states.

We used commercially available SmCo_5_ powders with particle sizes ranging from 1 to 10 μm (Fig. 1a). X-ray diffraction (XRD) showed that the material is single phase with a CaCu_5_-type hexagonal structure (Supplementary Fig. 1). Transmission electron microscopy revealed no grain boundaries in large particles ~ 10 μm, indicating that the individual particles are single crystals (Supplementary Fig. 2). The saturation magnetization of the powder at 2 T, measured with a superconducting quantum interference device (SQUID) magnetometer, is 100.2 A m^2^ kg^−1^ at room temperature (Supplementary Fig. 3). This value matches the reported saturation magnetization of SmCo_5_ (ref. ^20^), confirming that the particles are single crystalline. In this measurement, the loose particles were allowed to rotate and align themselves along the magnetic field. For all other magnetic measurements, the SmCo_5_ particles were fixed by using PVDF binder.

To control the absorption and desorption of hydrogen atoms in SmCo_5_ with external voltages, we used an electrochemical cell containing an aqueous electrolyte of 1 M KOH (Fig. 1b). The as-prepared SmCo_5_ electrode and Pt wires were the working and counter electrodes, respectively; the voltage of the working electrode was referenced to Hg/HgO. The process of voltage-controlled charging and discharging with hydrogen atoms can be described as follows. During the absorption (charging), the water molecules at the SmCo_5_/electrolyte interface were reduced into hydroxide and hydrogen atoms (reaction ① in Fig. 1b). Hydrogen atoms were first adsorbed onto the surface of the SmCo_5_ particles (*H*_ads_) and then, driven by the concentration gradient, diffused into the material and were absorbed in either tetrahedral or octahedral sites of the crystal structure (*H*_abs_, reaction ② in Fig. 1b). Conversely, during the desorption (discharging), the hydrogen atoms on the surface (*H*_ads_) were oxidized and removed, and then, driven by the gradient of concentration, H_abs_ diffused out, resulting in hydrogen desorption. As shown in the cyclic voltammogram (CV) curve (Fig. 1c), the two current peaks in the cathodic scan correspond to the adsorption and absorption of hydrogen atoms, respectively, whereas the current peak in the anodic scan relates to the desorption (or oxidation) of hydrogen atoms. The reversible absorption and desorption of hydrogen atoms are similar to that observed in the well-studied LaNi_5_ used for nickel-metal hydride batteries^23^.

The crystal structure of SmCo_5_ during the charging and discharging was monitored using in situ XRD measurements in transmission mode. The transmission mode allows the detection of the whole volume of the particles rather than only their surfaces. According to Fig. 1c, −1.2 and −0.4 V were applied to induce hydrogen absorption and desorption, respectively. When −1.2 V was applied, the 101 diffraction peak at 13.9° quickly diminished, and two split peaks appeared at 13.7°. The split peaks grew rapidly and remained stable after one hour, indicating that the whole sample was fully charged with hydrogen atoms (Fig. 1d). Mass spectrum confirmed that after charging in 1 M KOH in D_2_O, the intensity of deuterium peak increased significantly and became comparable to that of hydrogen peaks, confirming the absorption of hydrogen atoms in SmCo_5_ (Supplementary Fig. 4). The amount of the absorbed hydrogen was determined to be around 2.6 atoms per SmCo_5_ unit cell by thermogravimetric analysis (Supplementary Fig. 5). Rietveld analysis showed that upon hydrogen insertion the hexagonal CaCu_5_ structure expanded anisotropically in the basal plane with the c-axis nearly unaffected, revealing the distortion of the original hexagonal structure into an orthorhombic body-centered structure due to hydrogen insertion (Fig. 1b and Supplementary Fig. 6). When the voltage was changed to −0.4 V, the split peaks slowly diminished and the 101 peak started again to develop after ~ 8 h. After a prolonged time of discharging, the 13.9° peak was completely recovered, indicating that the discharging process was complete.

From the evolution of the XRD patterns, the subsequent stages of the charging and discharging processes can be inferred. When the fully charged sample (at −1.2 V for 1 h, sample ⓑ in Fig. 1d, e) was discharged at −0.4 V for 2 h, the diffraction patterns remained unchanged (sample ⓒ in Fig. 1d, e). This suggests that during the initial stage of the discharging process only the near-surface region of SmCo_5_ particle was depleted from hydrogen, forming a core-shell structure with the core containing hydrogen atoms (see schematics in Fig. 1e). Hereafter, the different states of the as-prepared SmCo_5_ sample (*S*_P_) after charging at −1.2 V for 1 h and further discharging at −0.4 V for 2 h and 40 h are referred to as fully charged (*S*_FC_), partially discharged (*S*_PDC_), and fully discharged (*S*_FDC_) samples, respectively (Fig. 1e).

### Voltage modulation of coercivity at room temperature

**Fig. 2:**
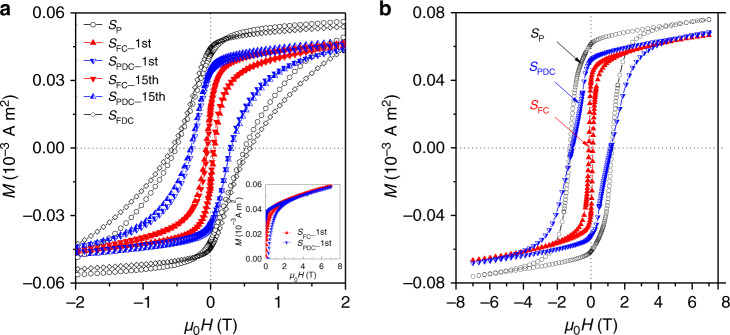
In situ voltage control of the coercivity in SmCo_5_ at room temperature. **a** Enlarged hysteresis loops of the as-prepared SmCo_5_ sample (*S*_P_) and those under the applied voltages of −1.2 V (*S*_FC_) and −0.4 V (*S*_PDC_). The *S*_P_ sample was repeatedly fully charged (at −1.2 V for 1 h, *S*_FC_) and partially discharged (at −0.4 V for 2 h, *S*_PDC_) for 15 times. Only the hysteresis loops after the 1st and the 15th charging and discharging cycles were shown, revealing excellent reversibility of the coercivity manipulation. The fully discharged sample (*S*_FDC_) was obtained by discharging the *S*_FC_ sample at −0.4 V for ~40 hours, showing the complete recovery of the coercivity to that of *S*_p_ sample. Inset in **a** shows the first quadrant of hysteresis loop of *S*_FC__1st and *S*_PDC__1st, showing no change of magnetization. **b** Hysteresis loops of the as-prepared SmCo_5_ sample (*S*_P_) and those under the applied voltages of −1.2 V (*S*_FC_) and −0.4 V (*S*_PDC_). In **a** the samples had particle sizes ranging from 1 to 10 μm; those in **b** were ball milled and had smaller particle sizes of ~1 μm.

We explored the response of the magnetic properties of SmCo_5_ to the applied voltages using in situ SQUID measurements. The coercivity of the *S*_P_ sample was ~0.5 T (Fig. 2a). After the sample was fully charged (*S*_FC__1st), its magnetization decreased by ~10%. More strikingly, the coercivity decreased by one order of magnitude to ~0.04 T. The observed reductions in the magnetization and coercivity are qualitatively consistent with previous results for RCo_5_H_x_ (R = rare-earth metal) synthesized in gaseous hydrogen^24,25^. When the sample was fully discharged (*S*_FDC_), both the magnetization and the coercivity of the sample were fully recovered to their initial values of the pristine (*S*_p_) sample (Fig. 2a and Supplementary Fig. 7).

Interestingly, we found that the short-time partial discharging can already significantly increase the coercivity. When *S*_FC_ was partially discharged at −0.4 V for 2 h (*S*_FC__1st→ *S*_PDC__1st in Fig. 2a), its magnetization remained nearly unchanged (inset in Fig. 2a). This is consistent with in situ XRD measurements detecting no change of the crystal structure (Fig. 1d, e). By contrast, the coercivity increased by a factor of seven from ~0.04 T to ~0.3 T. The reversibility of the coercivity was examined by alternately holding the sample at −1.2 V (1 h) and at −0.4 V (2 h) to fully charge and partially discharge the sample for 15 times. Magnetic measurements show that after the voltage-switching procedure, the hysteresis loops of both *S*_FC__15th and *S*_PDC__15th samples overlapped with those before, revealing an excellent reversibility of the voltage-modulation of coercivity in SmCo_5_ (Fig. 2a). Moreover, the voltage dependence of the coercivity in SmCo_5_ was studied by treating the individual *S*_P_ samples at various voltages from −0.9 V to −1.2 V and then to −0.4 V (Supplementary Fig. 8). As expected, the coercivity changed exactly at the voltages where the hydrogen absorption and desorption occur.

The modulation of the coercivity became larger in magnitude and faster in speed when using the SmCo_5_ powder with smaller particle sizes of ~1 μm, which displayed a high coercivity of ~1.2 T. After the full charging (*S*_FC_), the coercivity decreased by one order of magnitude to ~0.1 T. Astonishingly, when the *S*_FC_ was partially discharged, the coercivity (~1.1 T) was almost fully restored to that of the *S*_P_ sample (Fig. 2b). The modulation of coercivity between *S*_FC_ and *S*_PDC_ states can be repeated many times. The voltage-driven tuning of the coercivity thus reached an unprecedented value of ~1 T, more than two orders of magnitude larger than those achieved in ferromagnetic metals through charge doping^6,7,9^ and magneto-ionics^10–13^. Furthermore, the substantial shortening of the time for the complete recovery of coercivity compared with larger particles suggests that considerable further improvement in the speed can be achieved by reducing the particle sizes or using thin films.

### Voltage-assisted magnetization reversal at room temperature

**Fig. 3:**
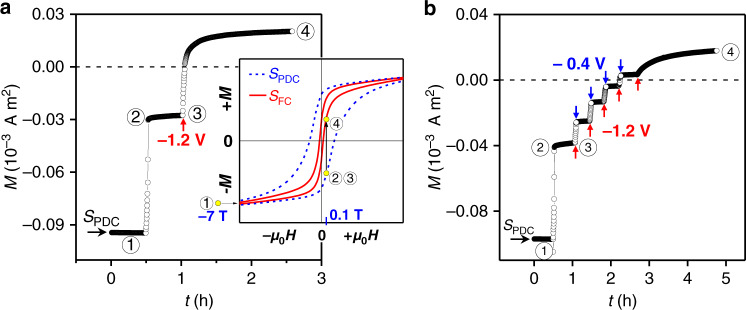
Voltage-assisted magnetization reversal in SmCo_5_ at room temperature. **a** Time evolution of the magnetization in the *S*_PDC_ sample with the voltages switched from −0.4 V to −1.2 V, showing the voltage-assisted magnetization reversal. Points ①, ②, ③, and ④ indicate different magnetization states as shown in the inset. **b** Time evolution of the magnetization in the *S*_PDC_ sample with the voltages switched repeatedly between −0.4 V and −1.2 V, showing the voltage-controlled quick and reversible “stop and reactivation” of the magnetization reversal. The red (blue) arrows denote the time points where −1.2 V (−0.4 V) was applied. Here, the same samples as in Fig. 2a were used, with particle sizes of 1–10 μm.

With the substantial modulation of the coercivity, the magnetization reversal can now be assisted by applying low voltages (Fig. 3a). In the measurement, we used the samples with particles sizes of 1–10 μm as those in Fig. 2a. First, we magnetized the *S*_PDC_ sample with a large magnetic field of −7 T (point ① in the inset). Then, the magnetic field was reversed to 0.1 T (point ②). As the 0.1 T field was smaller than the coercive field of the *S*_PDC_ sample (~0.3 T), the magnetization remained negative and nearly constant until the voltage was changed to −1.2 V (point ③). In response to the voltage change, the magnitude of magnetization decreased abruptly and changed from negative to positive in only ~3 min, showing the voltage-assisted magnetization reversal. After ~1.7 h, the magnetization became constant (④). Furthermore, the magnetization reversal can be stopped and reactivated on-demand by switching the applied voltages between −1.2 V and −0.4 V (Fig. 3b). The “stop and reactivation” process responded to the voltage switching within a few seconds and can be repeated many times without changing the magnetic field. The above results show that the giant modulation of coercivity and the assisted magnetization reversal can be achieved in micrometer-sized SmCo_5_ by electrochemically controlled hydrogen charging and discharging. Below the possible mechanisms are discussed, starting with the impact of the magnetocrystalline anisotropy constant (*K*_1_).

## Discussion

**Fig. 4:**
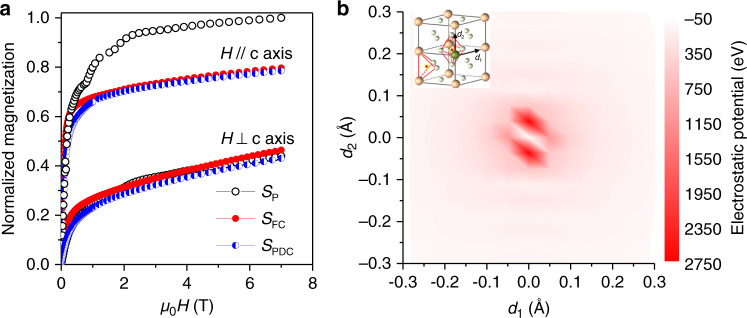
Dependence of the magnetocrystalline anisotropy of SmCo_5_ on the charging states. **a** Easy and hard axis magnetization curves of *S*_P_, *S*_FC_, and *S*_PDC_ samples. For comparison, the magnetization was normalized by the magnetization value of the *S*_P_ sample at 7 T along the easy axis. **b** Contour plot of the difference of the electrostatic potential around Sm^3+^ between SmCo_5_ and SmCo_5_H_3_ within (100) plane. Inset denotes the directions of *d*_1_ (along [010]) and *d*_2_ (along [001]) in hexagonal structure.

Figure 4a shows the easy and hard axis magnetization curves of the *S*_P_, *S*_FC_, and *S*_PDC_ samples after aligning and fixing the SmCo_5_ particles along their easy axis. The value of *K*_1_ was obtained by calculating the area enclosed between these two curves (Supplementary Fig. 9). After the full charging, the saturation magnetization (*M*_s_) of the *S*_P_ sample decreased by ~20%, while *K*_1_ decreased by ~40%. The decrease of *K*_1_ is qualitatively consistent with the results reported for LaCo_5_ and CeCo_5_ after gaseous hydrogenation^26^. Density functional theory (DFT) calculations confirmed that *K*_1_ decreased by ~30% when transforming from SmCo_5_ to SmCo_5_H_3_. Moreover, DFT calculations indicated that the decrease in *K*_1_ originates from the change of the electrostatic potential around the Sm^3+^ ions with the 4 f charge density essentially unchanged^27^ (Supplementary Fig. 10). After hydrogen insertion, the electrostatic potential increased more along the c-axis than along the b-axis (Fig. 4b). This partially canceled the original anisotropy of the electrostatic potential in the *S*_P_ sample, resulting in a decrease of both the crystal field and the *K*_1_ of the *S*_FC_ sample.

However, when the *S*_FC_ sample was partially discharged (*S*_PDC_), the values of *K*_1_ and *M*_S_ remained essentially unaffected, since the respective easy and hard axis magnetization curves of *S*_FC_ and *S*_PDC_ samples were nearly superimposed on each other (Fig. 4a). Generally, the coercivity of ferromagnetic materials is determined by the intrinsic magnetic parameters (*K*_1_ and *M*_s_) and the microstructural features, such as the orientation of grains, their sizes, and shapes^28^. During the migration of hydrogen atoms in interstitial sites, these microstructural features of the single-crystalline SmCo_5_ particles are expected to remain unaltered, and the change of coercivity should be attributed to the change of *K*_1_ and *M*_s_. However, the experimental results revealed the nearly unchanged *K*_1_ and *M*_s_ from the *S*_FC_ to *S*_PDC_ states (Fig. 4a). Since *K*_1_ and *M*_s_ were obtained by measuring the entire volume of the particle, this suggests that the coercivity is not controlled by the entire volume of the particle but rather by its near-surface region. As described earlier, when the *S*_FC_ sample was partially discharged, only the near-surface regions were actually affected (Figs. 1d, e and 2a). The ability to modulate the coercivity of the whole volume of the particle by only charging and discharging the near-surface region thus enables the kinetically fast control of coercivity, as verified by the fast reversible stop and reactivation of magnetization reversal in Fig. 3b. The responsible magnetization reversal mechanism regarding the near-surface region in determining the coercivity is next considered.

Because of the high magnetocrystalline anisotropy, the magnetization reversal in single-crystalline SmCo_5_ particles is controlled by the nucleation and growth of the reversed magnetic domains from surface defects where local gradient of *K*_1_ is capable of significantly lowering the nucleation field^29,30^. The nucleation field can be described by^31^

1 $$H_{\mathrm{n}} = \frac{1}{{2M_{\mathrm{s}}{\Delta}}}\left( {\gamma _{{\mathrm{SmCo}}_5} - \gamma _{{\mathrm{defect}}}} \right) - {\mathrm{DM}}_{\mathrm{s}}$$

in which Δ is the width of the transition region where the domain-wall energy changes from *γ*_defect_ at the defects to $\gamma _{{\mathrm{SmCo}}_5}$ in the crystal, and *D* is the local demagnetizing factor. Since the demagnetizing field (DM_*s*_) decreased slightly due to the reduced saturation magnetization (*M*_s_) after hydrogen insertion, the huge decrease in coercivity must be attributed to the decrease in the domain-wall energy gradient, $\left( {\gamma _{{\mathrm{SmCo}}_5} - \gamma _{{\mathrm{defect}}}} \right)/{\Delta}$. As shown above, *K*_1_ of the entire volume of the particle decreased by ~40% from the *S*_P_ to *S*_FC_ states (Fig. 4). It is reasonable to assume that the *K*_*1*_ of the near-surface region would decrease by the same amount when hydrogen insertion just started. By using $\gamma _{{\mathrm{SmCo}}_5} = 4{\mathrm{\surd AK}}_1$, in which *K*_1_ decreased by ~40% and the exchange constant (*A*) decreased by ~45%^26^, the domain-wall energy $\gamma _{SmCo_5}$ in the near-surface region was calculated to decrease by ~43% after hydrogen insertion. If ignoring the negligible value of *γ*_defect_, the value of $\left( {\gamma _{{\mathrm{SmCo}}_5} - \gamma _{{\mathrm{defect}}}} \right)$ would decrease by ~ 43%. Clearly, the magnetic softening due to the reduction of domain-wall energy is not enough to account for the observed 90% reduction of coercivity from *S*_P_ to *S*_FC_ sample. Additional reduction in the coercivity may originate from the widening of the transition region, Δ. In SmCo_5_ particles, its size is comparable to local defects, and, usually, it cannot be larger than 10–30 nm^31^. But after hydrogen insertion the inhomogeneous redistribution of hydrogen atoms around the surface defects can be more pronounced, and, consequently, the transition region can be significantly wider^32^. Moreover, when SmCo_5_ particles were charged with hydrogen atoms, the crystal structure changed from the hexagonal to the orthorhombic one and the unit cell volume expanded by ~5% (Fig. 1d). This may increase the mismatch between the surface defects and the surrounding region, broadening the transition region and reducing the coercivity. Exchange spring effect may also be involved at the soft core/hard shell interfaces. Yet, considering the gradual change of hydrogen concentration from the shell to the core as well as the rather small exchange correlation length of SmCo_5_ (2–4 nm), the exchange spring effect may be insignificant^33^.

In summary, using micrometer-sized SmCo_5_ as a bulk model material, we show that through electrochemically controlled insertion and extraction of hydrogen atoms in the metal structure, the bulk magnetic properties of ferromagnetic metals can be modulated with giant magnitudes. Our study offers an approach to overcome the limitations of the electric-field screening, opening the door to hugely and reversibly modify the bulk magnetic properties in ferromagnetic metals. This approach should be applicable to many rare earth-transition metal hard magnets, such as NdFeB^34^ and Sm_2_Co_17_ (ref. ^35^), as hydrogen diffusion in these materials has been normally observed. In application context, the ability to hugely tune their magnetic properties by applying small voltages, which has not been accessible before, will endow the ferromagnetic metals functions such as in magneto-electric actuation^36^, information storage, and processing^37,38^. For instance, the ability to reduce their coercivity temporarily will greatly reduce the energy to demagnetize and reverse the magnetization. Another probable application of our results lies in voltage-assisted magnetic storage. With its exceptionally high magnetocrystalline anisotropy (17.2 MJ m^−3^)^21^, SmCo_5_ can keep the magnetization stable against thermal agitation even when the bit size is 2–3 nm, pushing the area density up to 10 Tb/inch^2^. However, its use is hindered by the high coercivity_,_ which makes the writing of magnetic bits a problem^38^. The demonstrated ~1 T reduction of coercivity and the voltage-assisted magnetization reversal provides a promising approach to solve this problem, i.e. low-coercivity state for writing and high-coercivity state for long-term storage. It is anticipated that the hydrogen charging/discharging time can be significantly reduced when the material size is reduced to nanometer scale^39–41^ and the switching speed can be increased. The production of hydrogen atoms by electrochemical reduction of water molecules is considered much faster than the diffusion of hydrogen atoms in the material. We thus estimated the switching speed at the nanoscale by calculating the diffusion time according to the diffusion equation $l = \sqrt {Dt}$, in which *l* is the diffusion length, *D* the diffusion coefficient and *t* the diffusion time. In Fig. 1d, the charging/discharging time of SmCo_5_ particles with sizes of 1–10 μm is ~10 min/40 h. Therefore, the diffusion time for a thin film at the nanometer scale can be expected to be reduced by several orders of magnitudes to ms and sub-ms range, which is comparable to the fastest switching speed (~1 ms for 1-nm thick cobalt layer)^38^ achieved by magneto-ionics at similar length scales. In addition, based on this equation, the calculated diffusion coefficient falls in the range of 10^−8^–10^−13^ cm^2^ s^−1^ at room temperature, still much smaller than that obtained in gaseous hydrogen (10^−8^–10^−10^ cm^2^ s^−1^)^42,43^, indicating that significant improvements in switching speed may be achieved by optimizing the electrochemical-cell (device) geometry^44,45^. Furthermore, the diffusion of hydrogen atoms can be speeded up significantly by including high diffusion paths such as grain boundaries. This could be especially exciting for use in neuromorhpic computing, where the slow switching rate of ~100 Hz and the large tuning magnitude are needed^46^.

## Materials and methods

### Materials and microstructure characterization

The SmCo_5_ powders were purchased from Alfa Aesar (Stock No. 42732.18). The composition of the powder was analyzed by inductively-coupled plasma mass spectroscopy (Supplementary Table 1) and its microstructure was characterized using field-emission scanning electron microscope (Zeiss Ultra 600), powder X-ray diffraction with a Mo K_α_ source (Philips X’Pert Analysis) and transmission electron microscope (FEI Titan 80-300). The preparation of TEM samples followed the ordinary procedure of cutting, lifting and milling using FIB/SEM system (FEI Strata 400 and Zeiss Auriga 60). For the magnetic measurements in Fig. 2b, the as-received powder was vibration-milled for 1 h (Retsch MM400) and sieved to reduce the particle size. For all other measurements, the as-received powders were used.

### Preparation of the SmCo5 electrode and the electrochemical set-up

To prepare the SmCo_5_ electrode, the SmCo_5_ particles were mixed with PVDF solution to form slurry, which was then coated onto thin copper foils (thickness ~15 μm). The slurry/Cu foil composite was dried at 80 °C for 4 h, and afterward compressed under a pressure of ~100 MPa to further fix the particles and to increase the electrical conductivity between SmCo_5_ particles and the Cu foil. We prepared the PVDF solution by dissolving PVDF powder in NMP solution at a mass ratio of 5:95 with overnight stirring.

The charging and discharging of the SmCo_5_ electrodes were carried out under potentiostatic control in a three-electrode electrochemical system (Autolab PGSTAT 302N). The working, counter, and reference electrodes were the SmCo_5_ electrode, Pt wires and a pseudo Ag/AgCl electrode, respectively. The potential of the peuso Ag/AgCl electrode is 0.300 ± 0.002 V more positive than the standard Hg/HgO (1 M KOH) electrode, and for comparison, all the voltages in the paper were converted to the Hg/HgO scale. The electrolyte was an aqueous electrolyte of 1 M KOH prepared from ultrapure water with a resistivity of ~18.2 MΩ.

### In situ XRD measurement

The crystal structure of the SmCo_5_ electrode under the application of −1.2 V and −0.4 V was monitored by in situ XRD with a parallel beam laboratory rotating anode diffractometer (Mo K_α_ radiation) in transmission geometry. The transmission geometry allowed the detection of the entire volume of the SmCo_5_ particles rather than only their surfaces. For in situ measurement, the SmCo_5_ electrode, as the working electrode, was attached to a glass plate (thickness ~0.1 mm) and then immersed in the 1 M KOH electrolyte contained in plastic bags. The counter and reference electrodes were the Pt wire and the pseudo Ag/AgCl electrode, respectively. Diffraction patterns were collected every 371 seconds with a Pilatus 300K-W area detector. The function NIST SRM660b LaB_6_ powder was used for the detector calibration and determination of the instrumental resolution. The 2D diffraction images were integrated using the pyFAI software and analyzed with the Rietveld method (TOPAS V6). The isostructural orthorhombic β^II^ structure of PrCo_5_H_3_ (*Im*2*m* space group symmetry) was used as a structure model for the SmCo_5_ after hydrogen insertion.

### In situ SQUID measurement

In situ magnetic measurement was carried out with a custom-built miniaturized Teflon electrochemical cell in a superconducting quantum interference device (SQUID, MPMS3) at room temperature. In the electrochemical cell, the SmCo_5_ electrode, Pt foil and peuso Ag/AgCl electrode were the working, counter and reference electrodes, respectively. The electrolyte was 1 M KOH. The SmCo_5_ electrode and the Pt foil were attached to the flat surface of a plastic rod, while the reference electrode was threading through a capillary. The magnetic measurements were performed at the sealed mode of SQUID. All magnetic measurements were performed with the applied magnetic field parallel to the surface of the Cu foil.

For the determination of magnetocrystalline anisotropy constant (*K*_1_), the SmCo_5_ particles were first aligned in a homogeneous magnetic field before the drying of the slurry/Cu foil composite. Other steps in the preparation of the SmCo_5_ electrode were the same as those described earlier. Before the magnetic measurements in SQUID, the SmCo_5_ electrode was demagnetized with the vibrating fields from a value of 7–0 T. The sample was first measured along the easy axis. Then, the sample was removed from the plastic rod and remounted in a perpendicular direction and measured again. According to ref. ^37^, *K*_1_ was calculated by integrating the area enclosed between the hard and easy axis magnetization curves. Since the applied magnetic field was way smaller than the anisotropy field of SmCo_5_ (~40 T), these two curves were extrapolated until they met and then the enclosed area was calculated.

### TG and APT measurement

Thermogravimetric (TG) measurement was conducted in a Sensys Evo TG-DSC apparatus (Setaram). The as-prepared SmCo_5_ electrode was fully charged at −1.2 V for 1 h. To remove the residual water, the fully charged sample was rinsed into dehydrated acetone several times, and transferred into the TG chamber after the drying for the TG measurement. To analyze the evolved gas, mass spectrometry was carried out simultaneously with an OmniStar (Pfeiffer, Germany). During the measurements, the temperature was ramped at a rate of 5 °C min^−1^ to 80 °C and then held for another 2 h.

For the atom probe tomography (APT) measurement, the SmCo_5_ electrode was charged at −1.2 V for 1 h in 1 M KOH in D_2_O using the three-electrode system as described earlier. After the full charging, the sample was transferred to FIB/SEM system for the cutting, milling and lifting at room temperature (Zeiss Auriga 60). To refine the APT tip, annular milling was used to create the needle-shaped morphology with a diameter less than ~100 nm. APT measurements were conducted on a CAMECA-LEAP 4000×HR instrument in laser pulse mode (wave length 355 nm, pulse frequency 100 kHZ, pulse energy 60 pJ, evaporation rate 0.50%) at a specimen temperature of 20 K. APT reconstruction and analysis were carried out using the CAMECA IVAS version 3.6.1 software.

### DFT calculation

DFT calculations were performed using all electron full potential local orbital (FPLO) code version 18.00-52 (ref. ^47^). The exchange-correlation energy functional was approximated using the generalized gradient approximation within Perdew−Burke−Ernzerhof parameterization^48^. A linear tetrahedron method was used for the *k*-space integration with Blöchl corrections. *k*-point meshes of 8 × 8 × 10 and 10 × 10 × 10 were used for the SmCo_5_ and SmCo_5_H_3_ samples, respectively. The 4f electrons of Sm^3+^ have been treated within atomic limit approach (LSDA + U). The magnetocrystalline energies (MAE) were calculated using the full relativistic mode. After checking the dependence of MAE on U, the desired MAE was obtained with *U* = 8 and 5 eV for SmCo_5_ and SmCo_5_H_3_, respectively. The lattice constant and atomic positions were relaxed for SmCo_5_ and SmCo_5_H_3_. The optimized lattice constants and volume agreed well with the experimental values within a discrepancy of ~1%.

## Supplementary information

Supplementary Information

Peer Review File

## Data Availability

All data needed to evaluate the conclusions of the study are present in the paper or the supplementary materials.
