# Supplementary material for: Giant voltage-induced modification of magnetism in micron-scale ferromagnetic metals by hydrogen charging
Source: Nat Commun. 2020 Sep 24;11:4849. doi: 10.1038/s41467-020-18552-z (PMC7519083; doi:10.1038/s41467-020-18552-z)
Supplement: Supplementary file 1 — Supplementary Information [file 41467_2020_18552_MOESM1_ESM.pdf]

Supplementary information

Giant Voltage-Induced Modification of Magnetism in Micron-Scale Ferromagnetic Metals by Hydrogen Charging

Ye et al.

This PDF file includes:

Supplementary Figure 1 to 10

Supplementary Table 1

Figures and figure captions

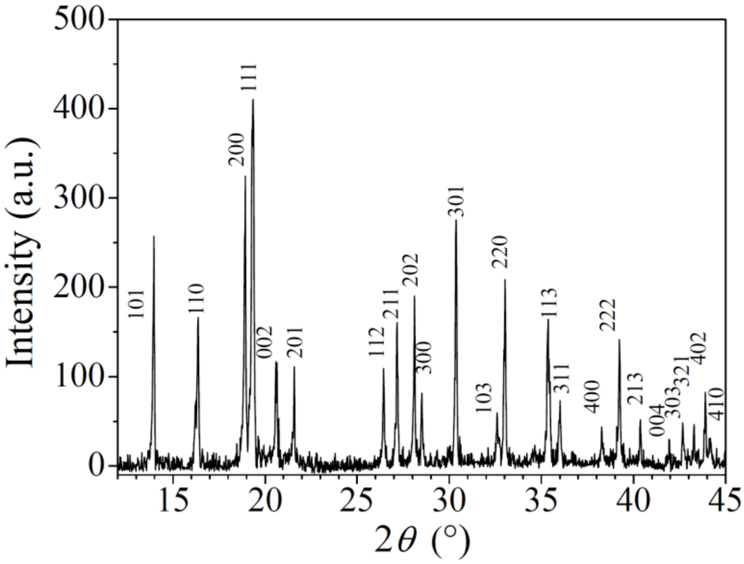

Supplementary Figure 1 An XRD pattern of the as-received $SmCo_5$ powder.

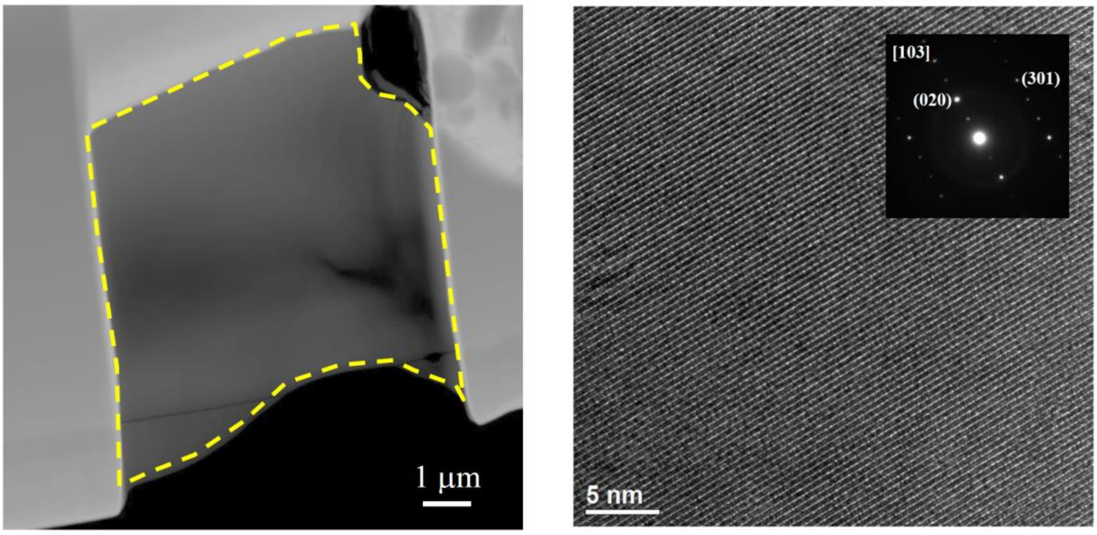

Supplementary Figure 2 (A) A dark-field TEM image of the $SmCo_5$ particle with a size ~ 10 μm, revealing no grain boundaries inside the particle. **(B)** The corresponding high-resolution TEM (HRTEM) image with a selected area diffraction pattern (inset).

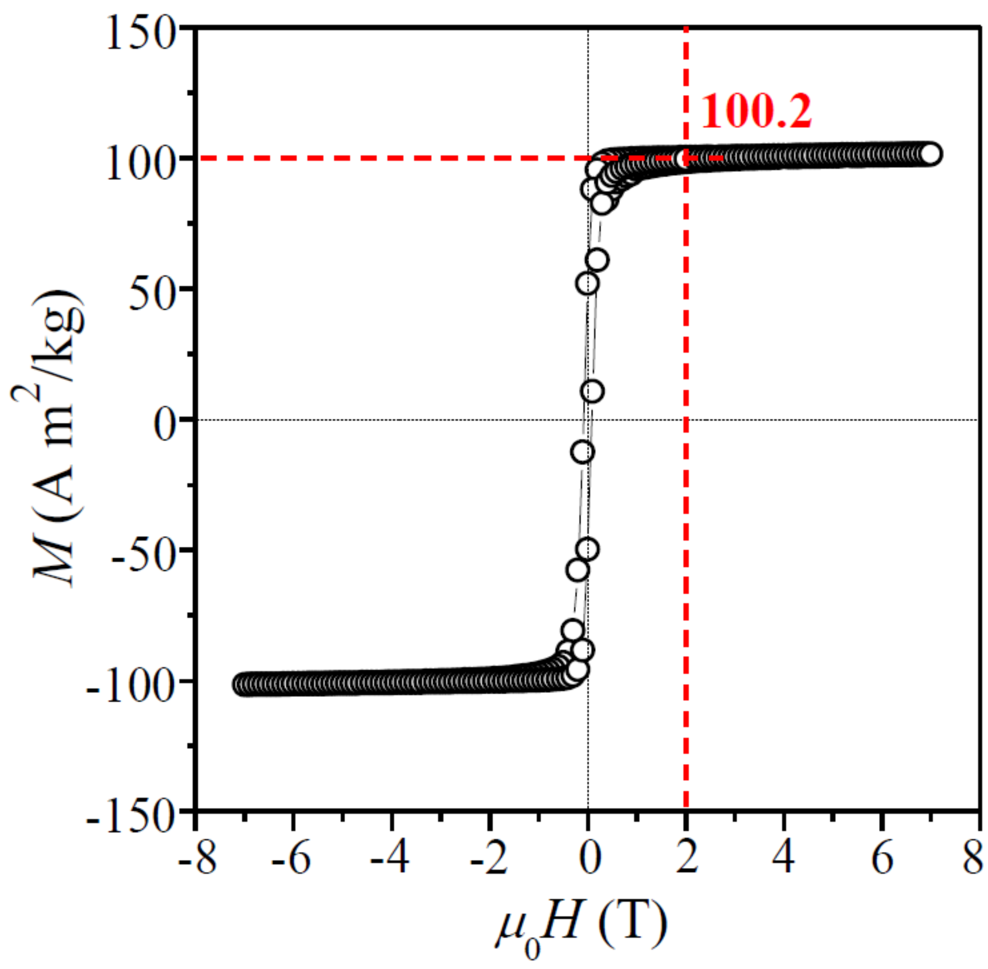

Supplementary Figure 3 Magnetic hysteresis loop of the as-received $SmCo_5$ powder. In the measurement, the particles were kept in a capsule (4096-388, Quantum Design) and allowed to rotate and align themselves along the magnetic field.

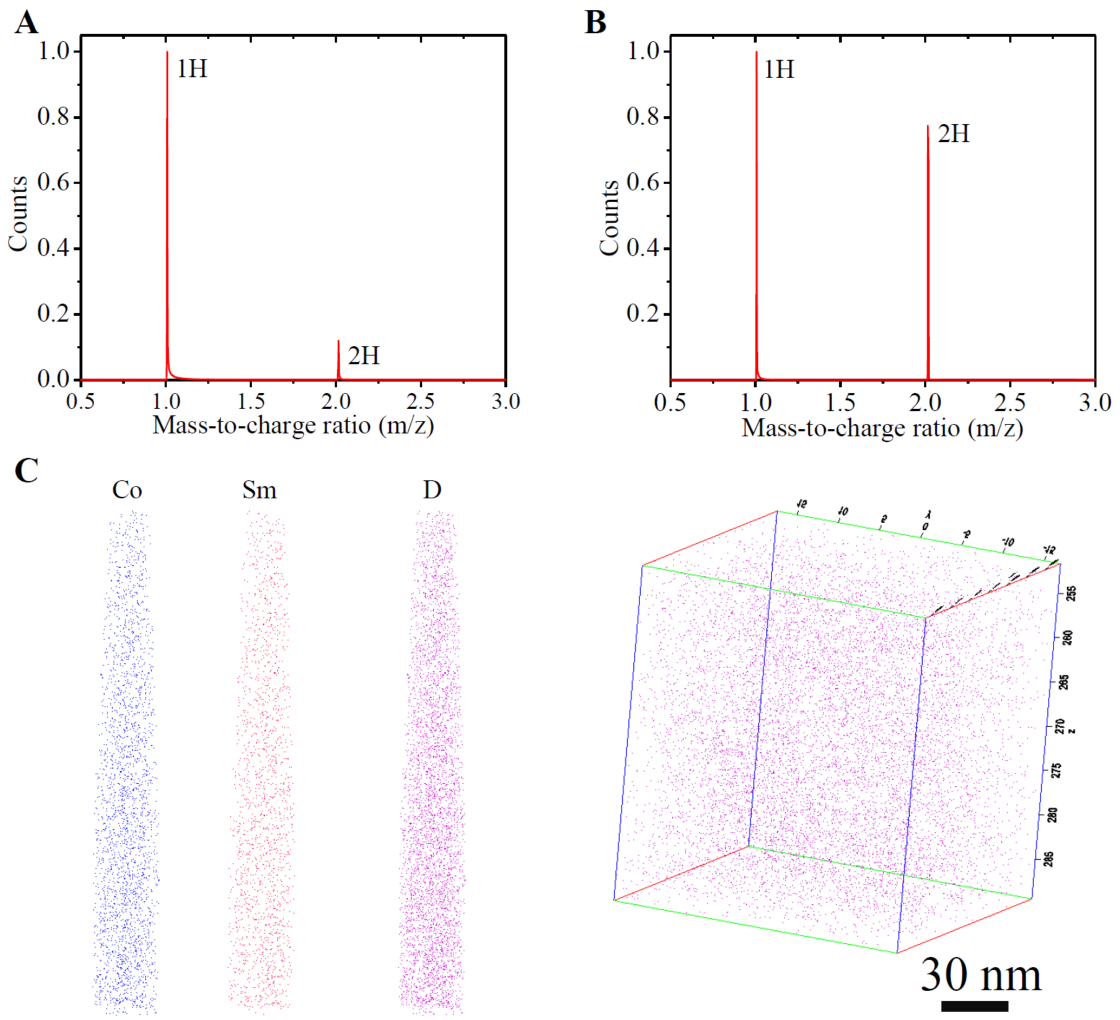

Supplementary Figure 4 The mass spectrums of (**A**) the as-prepared $SmCo_5$ sample and (**B**) the fully-charged sample in 1 M KOH in D_2O, showing that the concentration of ^2H (deuterium) increased significantly and became comparable to that of ^1H after the charging. Here, the fully-charged sample (S_{FC}) was obtained by holding the as-prepared sample (S_P) at -1.2 V for 1 hour in 1 M KOH in heavy water (D_2O). (**C**) 3D atomic reconstruction of the S_{FC} sample with deuterium atoms.

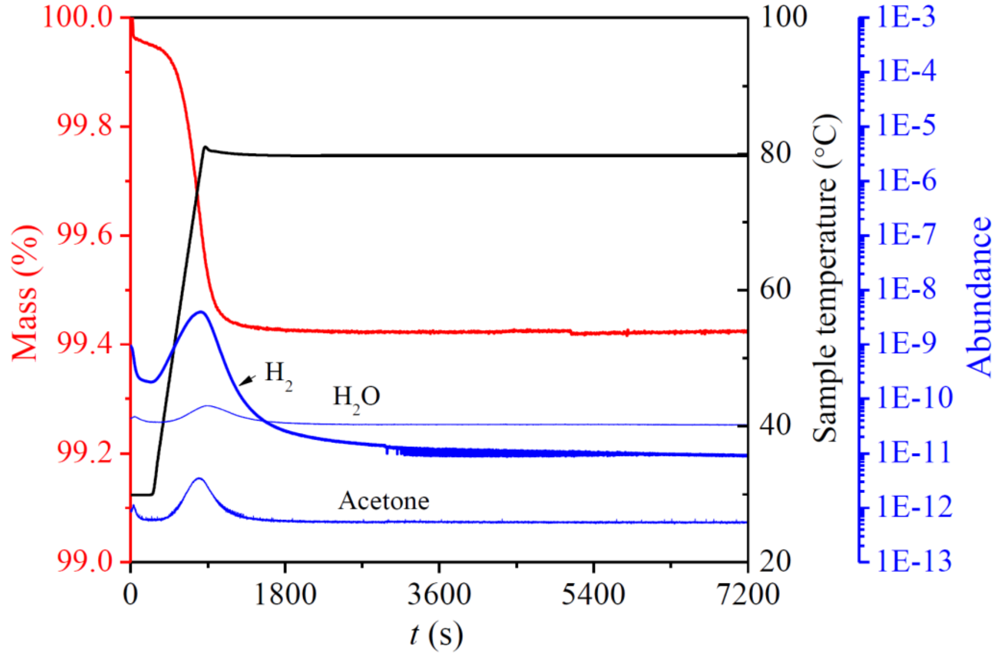

Supplementary Figure 5 Time evolution of the sample mass (S_{FC}) and the mass spectrums of hydrogen gas, water and acetone during the thermogravimetric analysis of the fully-charged sample (S_{FC}). The ramp rate of temperature was 5 ℃/min. The concentration of hydrogen atoms was roughly estimated at 2.6 atoms per $SmCo_5$ unit cell.

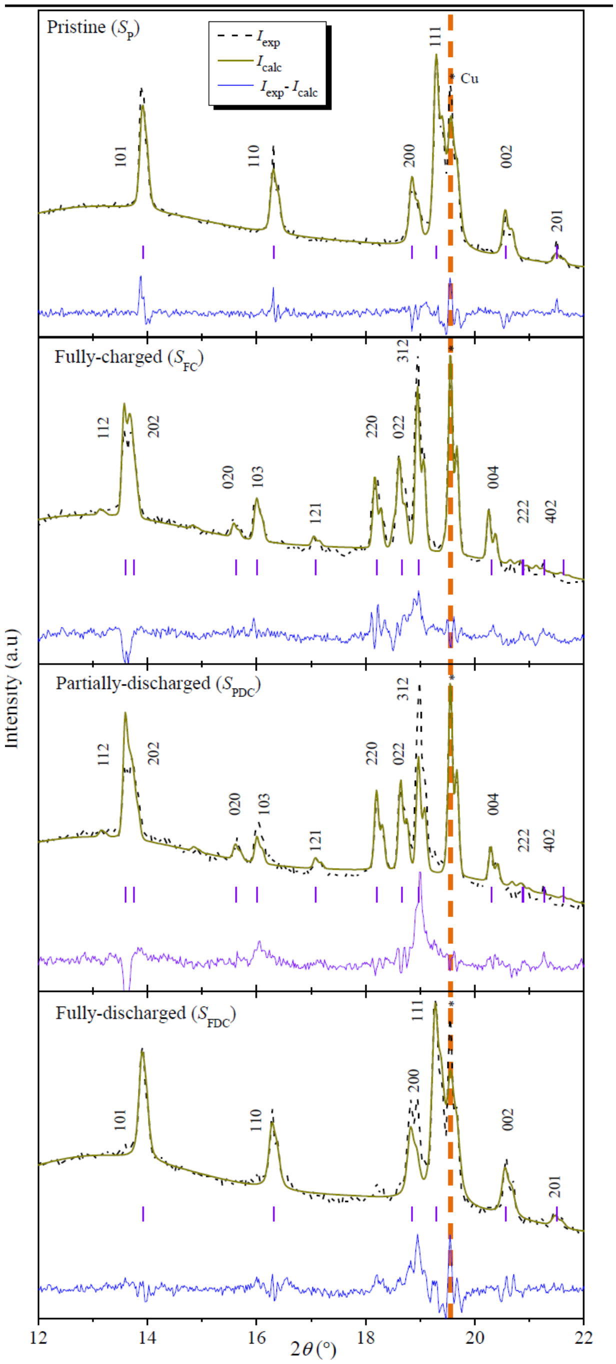

Supplementary Figure 6 Rietveld refinement results of the S_P, S_{FC}, S_{PDC} and S_{FDC} samples. After the full charging, the $SmCo_5$ phase transformed completely from the original hexagonal structure (lattice parameters: a_h = 5.001(1) Å, c_h = 3.972(3) Å) to an orthorhombic structure with space group symmetry *Im*2*m* (lattice parameters: a_o = 8.749(2) Å, b_o =5.219(1) Å, c_o = 8.049(2) Å). This phase transformation was accompanied by a volume expansion of ~5%. When the material was fully discharged, the structure reverted to the original hexagonal structure.

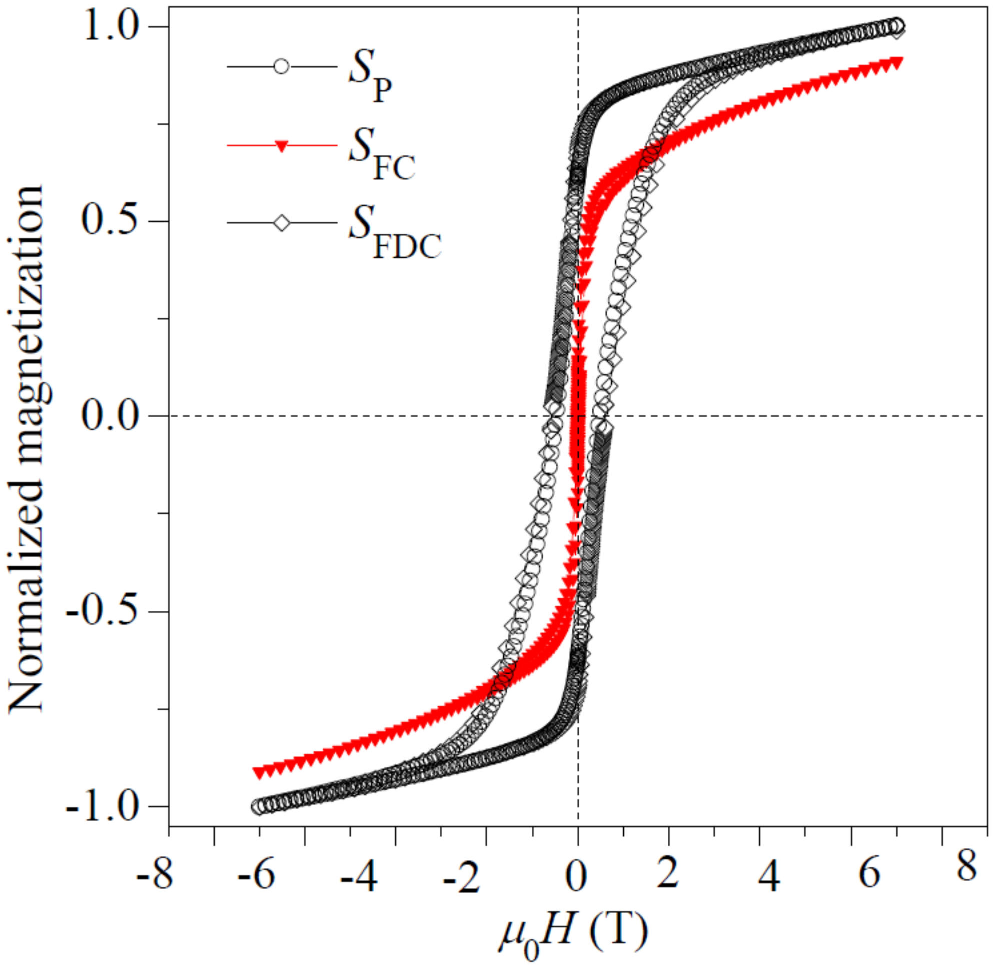

Supplementary Figure 7 Magnetic hysteresis loop of the S_P, S_{FC} and S_{FDC} $SmCo_5$ samples, showing the full recovery of the coercivity and the magnetization after the full discharging. All the magnetization values have been normalized with respect to the magnetization value of the S_P sample at 7 T.

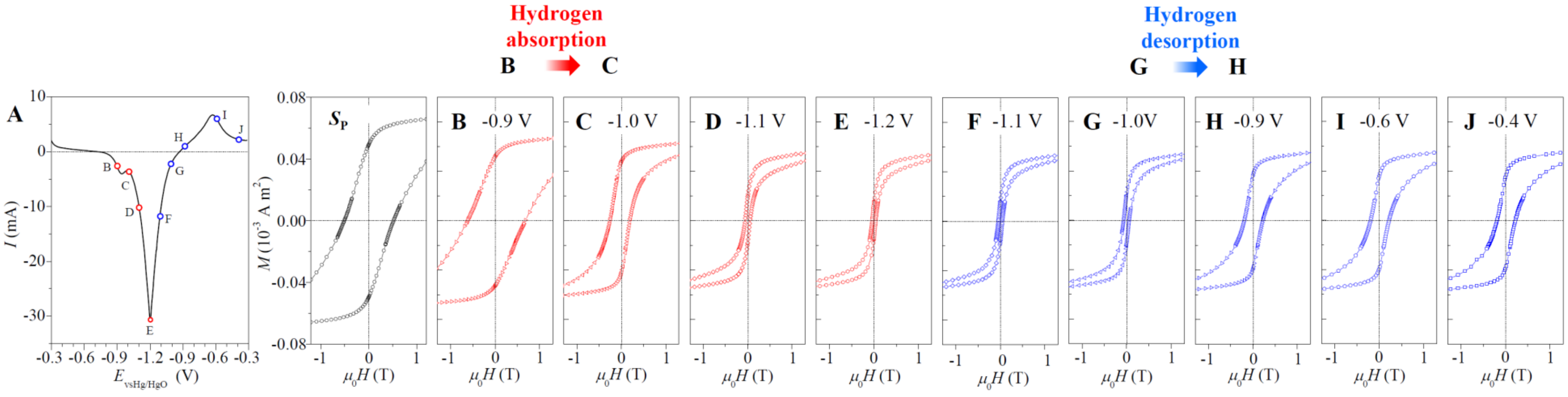

Supplementary Figure 8 Voltage dependence of coercivity in $SmCo_5$ at room temperature. (**A**) Replot of the CV curve of the $SmCo_5$ electrode shown in Fig. 1C, which lists a series of voltages used for the voltage treatment. The samples B, C, D and E were obtained by subjecting the individual S_P samples to 1 hour of charging at -0.9 V, -1.0 V, -1.1 V and -1.2 V, respectively, whereas samples F, G, H, I and J were obtained by subjecting the individual S_{FC} samples to 1 hour of discharging at -1.1 V, -1.0 V -0.9 V, -0.6 V and -0.4 V, respectively. (**B**)-(**J**) Enlarged magnetic hysteresis loops of the corresponding samples indicated in (**A**). It was obvious that the coercivity only started to decrease with the voltages decreased below -1.0 V (**B** to **C**), the threshold voltage of hydrogen absorption. In the reversed direction, the coercivity only started to increase abruptly as the voltage exceeded -0.92 V (**G** to **H**), the threshold voltage of hydrogen desorption.

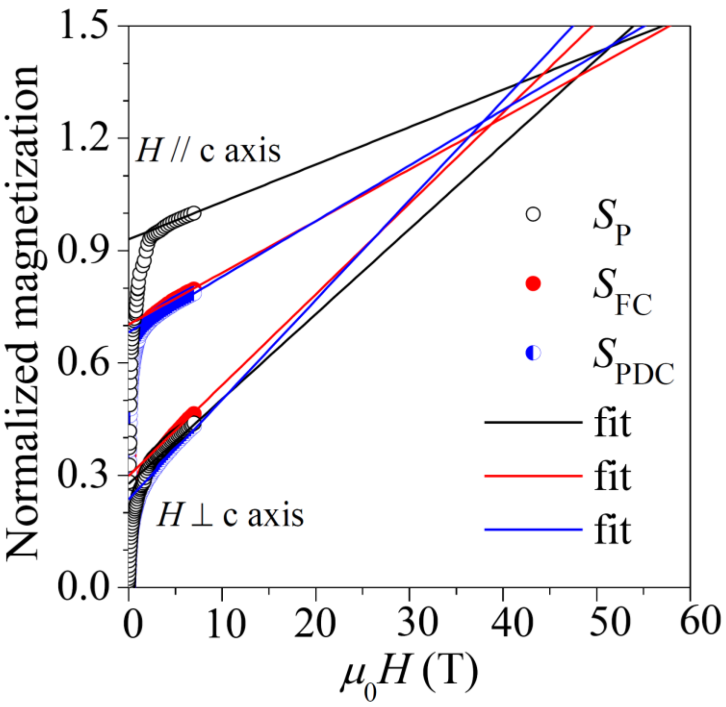

Supplementary Figure 9 The easy and hard axis magnetization curves of the S_P, S_{FC} and S_{PDC} samples. For comparison, the magnetization is normalized by the magnetization value of the S_P sample at 7 T along the easy axis. To compare the magnetocrystalline anisotropy, we first extrapolated the easy axis and hard axis curves until they met and then calculated the area enclosed between them.

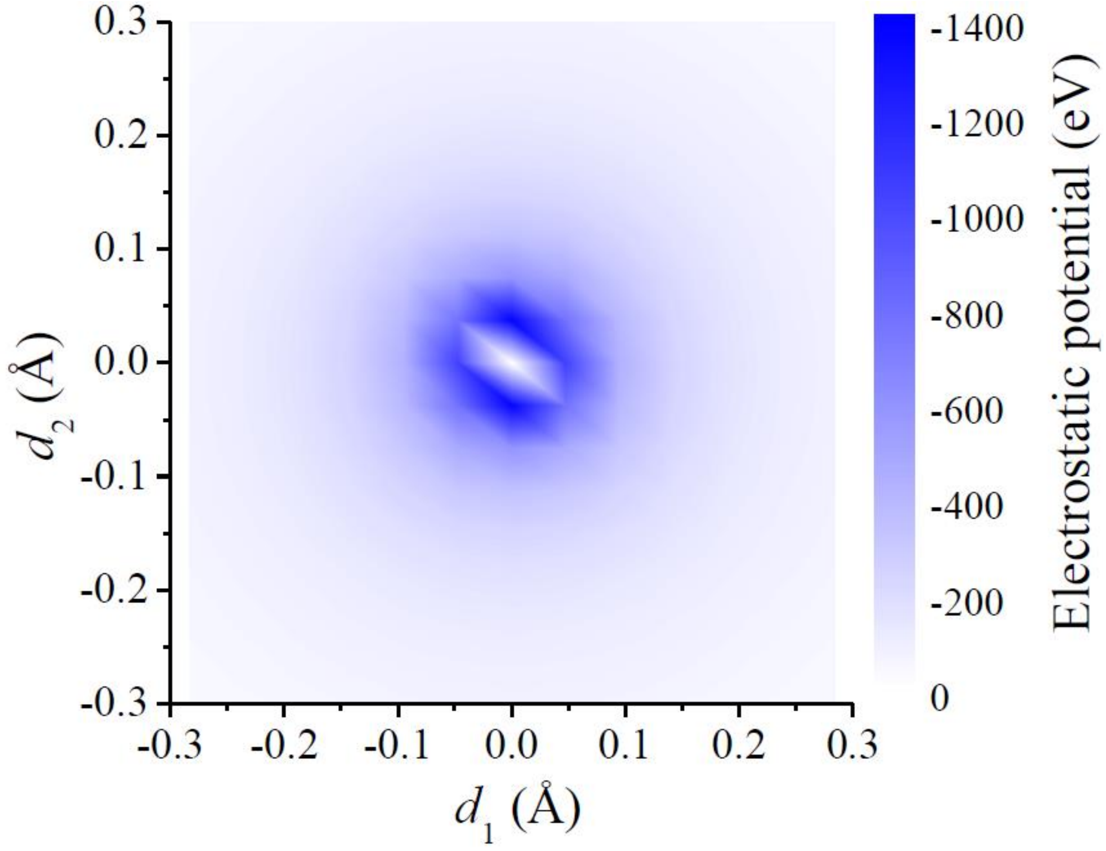

Supplementary Figure 10 Contour plot of the electrostatic potential around Sm^{3+} for the $SmCo_5$ sample (S_P) within (100) plane.

	Fe	Co	Ni	Cu	Zr	Sm
Composition (wt. %)	0.0298	64.9	0.0234	0	0	32.5

Supplementary Table S1 Chemical composition of the as-received SmCo5 powder probed by inductively coupled plasma mass spectrometry (ICP-MS).
